# Unraveling Intestinal Microbial Shifts in ESRD and Kidney Transplantation: Implications for Disease-Related Dysbiosis

**DOI:** 10.3390/microorganisms11112747

**Published:** 2023-11-10

**Authors:** Pengpeng Yan, Sulin Luo, Luying Guo, Xingxia Wang, Xue Ren, Junhao Lv, Ying Chen, Xinyu Lin, Jianghua Chen, Rending Wang

**Affiliations:** 1 Kidney Disease Center, The First Affiliated Hospital, College of Medicine, Zhejiang University, Hangzhou 310003, China; yanpengpeng@zju.edu.cn (P.Y.); luo18723557142@163.com (S.L.); guoluying@zju.edu.cn (L.G.); 18767124535@163.com (X.W.); mailrenxue@163.com (X.R.); lvjunhao@zju.edu.cn (J.L.); cy-zyb@163.com (Y.C.); 15824419378@163.com (X.L.); chenjianghua@zju.edu.cn (J.C.); 2 Key Laboratory of Kidney Disease Prevention and Control Technology, Hangzhou 310003, China; 3 National Key Clinical Department of Kidney Diseases, Hangzhou 310003, China; 4 Institute of Nephrology, Zhejiang University, Hangzhou 310003, China; 5 Zhejiang Clinical Research Center of Kidney and Urinary System Disease, Hangzhou 310003, China; 6 Department of Nephrology, 903rd Hospital of PLA, Hangzhou 310013, China; 7 Department of Nephrology, Huzhou Central Hospital, Huzhou 313000, China

**Keywords:** gut microbiome, dysbiosis, end-stage renal disease, kidney transplantation, borderline change, 16S rRNA sequencing

## Abstract

The composition of the gut microbiome is profoundly influenced by the accumulation of toxins in end-stage renal disease (ESRD) and specific medical treatments during kidney transplantation (KT). However, variations in results may arise due to factors such as genetics, dietary habits, and the strategy of anti-rejection therapy. Therefore, we conducted a 16S rRNA sequencing study to characterize intestinal microbiomes by using 75 fecal specimens obtained from 25 paired Chinese living donors (LDs) of kidneys and recipients before and after KT. Surprisingly, similar enterotypes were observed between healthy LDs and ESRD recipients. Nonetheless, following KT, the fecal communities of recipients exhibited distinct clustering, which was primarily characterized by Escherichia–Shigella and Streptococcus at the genus level, along with a reduction in the diversity of microbiota. To further explore the characteristics of gut microorganisms in early rejection episodes, two recipients with biopsy-proven borderline changes during follow-up were enrolled in a preliminary sub-cohort study. Our findings reveal a comparable construction of gut microbiota between ESRD patients and their healthy relatives while also highlighting the significant impact of KT on gut microbial composition.

## 1. Introduction

The human gut microbiota, a vast population of commensal microorganisms residing within our gastrointestinal tract, have emerged as a focal point of research due to advancements in high-throughput biotechnology for the analysis of microbial communities, such as metagenomics [1] and 16S rRNA sequencing [2]. Previous research has revealed a bidirectional interaction between gut microbes and the host immune system that influences the process of host infections, immune disorder, and allograft tolerance [3,4,5].

In transplant cohorts, the post-transplant bacterial enterotypes undergo significant disruption due to the routine administration of immunosuppressive drugs, antimicrobial agents [6], and proton pump inhibitors (PPIs) [7], thereby exerting a considerable impact on the survival of both grafts and recipients [8,9]. Recent investigations have delved into the role of gut microbiota in the clinical course of kidney transplantation (KT), primarily focusing on the Caucasian/African-American/Hispanic population. For instance, Fricke et al. conducted a longitudinal study of rectal microbial communities during the KT period, revealing notable changes predominantly within the phylum *Firmicutes* [10], whereas Lee et al. observed a pronounced elevation in the phylum *Proteobacteria* instead of *Firmicutes* in a relatively small KT cohort [11]. While Guangren et al. observed a significant reduction in the abundance of the genera *Bacteroides*, *Megamonas*, and *Prevotella* in East Asian KT recipients [12], their study enrolled hemodialysis patients as controls, which may have introduced potential inter-individual differences, especially considering the influence of dietary patterns and the inherent variability in the gut microbiota among different populations [13]. Crucially, the intricate interplay among gut microorganisms, their accompanying microbial metabolites, and the host’s metabolic processes exerts a profound influence on the host’s immune system and overall health, particularly in the context of post-KT rejection events and post-transplant opportunistic infections. Fourteen microbial metabolites, including *N*-Palmitoylsphingosine, Enoxolone Arg-Glu, and Methylguanidine, were identified as discriminatory markers for antibody-mediated rejection (ABMR) in KT recipients [14]. Additionally, the persistence of gut bacterial translocation after KT was linked to the downregulation of serum inflammation biomarkers, including sCD14, which exhibited a negative correlation with acute rejection following transplantation [15]. A loss of butyrate-producing bacteria was noted in KT recipients in comparison to healthy controls [7], and a lower relative abundance of this microorganism has been linked to an increased risk of respiratory viral infections [16]. *Enterococcus* is apparently enriched in patients who experience *Enterococcus*-related urinary tract infections [11]. Even in cases of noninfectious post-transplant diarrhea, 13 downregulated commensal genera have been identified [17]. With the development of in-depth profiling in intestinal bacterial communities following KT, the strategy of manipulating the structure of the gut microbiome holds promise as a potential clinical therapy for enhancing the biocompatibility of renal allografts. Wu et al. demonstrated the efficacy of an intervention involving a high-fiber diet in preventing intestinal dysbacteriosis, resulting in reduced evidence of rejection and prolonged survival in a murine KT model [8]. 

Despite the growing volume of research, there remains a considerable knowledge gap concerning the alterations in the gut microbiome and metabolomics following KT, especially in individuals of East Asian descent. To preliminarily bridge this gap, we conducted a 16S rRNA sequencing analysis of 75 fecal specimens from 25 paired Chinese KT recipients and living donors (LDs) to investigate the gut dysbiosis of patients with end-stage renal disease (ESRD) and explore microbial population shifts following allogenic KT, with a specific focus on graft outcomes. Notably, we employed samples from the relatives of ESRD patients and the patients themselves before transplantation as controls in our investigation of the intestinal microbial shifts in ESRD and KT. This deliberate choice was intended to mitigate the potential impacts of dietary patterns and inherent variability in our study.

## 2. Materials and Methods

### 2.1. Transplant Cohort

A total of 50 participants were enrolled between April 2019 and July 2019, with 25 living donors (LDs) and 25 paired ESRD patients who received their first KT at our center. Fecal specimens were collected from the donors and intended recipients one day before the surgery. Additionally, one fecal sample was obtained from each allograft recipient in the first 2 months (±1 week) after transplantation, when the serum creatinine level reached a steady state, and a close clinical follow-up was carried out during this period. This study was approved by the Research Ethics Committee of the First Affiliated Hospital, College of Medicine, Zhejiang University (#2019-21). Written informed consent was obtained from each participant upon enrollment.

### 2.2. Sub-Cohort Selection

To investigate the alterations in the gut microbiota in severe chronic kidney disease patients compared to healthy controls, we developed *Cohort 1* to compare the difference in microbial communities between the healthy LDs (LD group, *n* = 25) and the paired pre-transplant ESRD recipients (preT_R group, *n* = 25).

Subsequently, specimens from the same recipients after KT (post-transplant recipients, postT_R, *n* = 25) were involved in *Cohort 2* to identify the dynamic transformation of microbial populations owing to the transplantation and medical treatment. Induction therapy comprised basiliximab (96%) or ATG (anti-thymoyte globulin) (4%). All KT recipients received a consistent immunosuppression treatment consisting of a combination of triple regimens that included steroids, tacrolimus, and mycophenolate mofetil. To prevent cytomegalovirus infection and pneumocystis pneumonia (PCP), 88% of recipients received ganciclovir and sulfamethoxazole/trimethoprim during the study period, while 12% discontinued this treatment due to creatinine elevation. All recipients were initially treated with 5 days of intravenous PPIs after KT, followed by 1 month of oral PPIs, and they were then switched to teprenone capsules. No death or renal graft failure, which was defined as the initiation of continuous dialysis therapy, was observed during the follow-up period.

**Figure 1:**
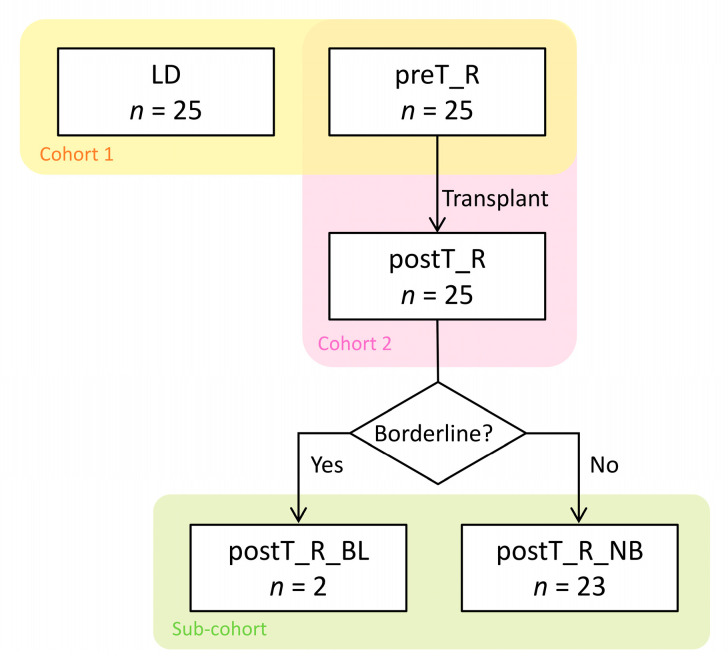
Study design and flow diagram. In *Cohort 1*, a total of 50 fecal samples were collected from 25 living donors (LDs) and 25 paired allograft recipients diagnosed with ESRD before the kidney transplant (KT) surgery (pre-transplant recipients, preT_R) to compare the construction of the gut microbiota in ESRD patients and their healthy relatives (yellow background). In *Cohort 2*, another 25 fecal specimens were gathered from the same recipients in the first 2 months (±1 week) after transplantation (post-transplant recipients, postT_R) to study the dynamic alterations in the microbial population owing to KT and medical treatment (pink background). Subsequently, two recipients received an allograft biopsy within 6 months after KT and were proven to experience borderline change (BL) for acute T-cell-mediated rejection; thus, a Sub-cohort was enrolled to identify the potential shifts in microbiota during early rejection episodes (green background).

To further study the shift in the gut bacterial spectrum during early rejection episodes, a Sub-cohort was selected from the postT_R group based on allograft-biopsy-proven borderline change (BL) for acute T cell-mediated rejection (TCMR) according to the Banff 2019 criteria [18] within six months after KT. In this chart review sub-cohort, the participants were divided into two groups: the BL recipients (postT_R_BL, *n* = 2) and the non-BL recipients (postT_R_NB, *n* = 23). A diagram illustrating the above selection process is presented in Figure 1.

### 2.3. Fecal Specimen Collection and DNA Extraction

Based on the above cohort, 75 fecal specimens were prospectively collected for subsequent 16S rRNA deep sequencing. Each stool sample was aliquoted into approximate 200 mg portions and stored at −80 °C in 1.5 mL of RNase-free sterile tubes (Axygen, Union City, CA, USA). DNA was extracted from the frozen fecal aliquot using the E.Z.N.A.^®^ Stool DNA Kit (#D4015, Omega, New York, NY, USA) according to the manufacturer’s instructions. The DNA quality was further assessed using agarose gel electrophoresis and quantitated using NanoDrop (Thermo Scientific, Waltham, MA, USA). 

### 2.4. 16S rRNA Deep Sequencing

The amplification of extracted DNA samples, the construction of DNA libraries, and the deep sequencing of nucleotides were performed on the NovaSeq PE250 platform by LC-Bio Technology Co., Ltd., Hangzhou, China. Briefly, the hypervariable V3–V4 region of the 16S rRNA gene was amplified via PCR using primers 341F (5′-CCTACGGGNGGCWGCAG-3′) and 805R (5′-GACTACHVGGGTATCTAATCC-3′) [19] in a final volume of 25 μL of the reaction mixture, which contained 12.5 μL of Phusion Hot Start Flex 2X Master Mix (#M0536L, NEB, Ipswich, MA, USA), 25 ng of template DNA, 2.5 μL of each primer, and appropriate PCR-grade water, with multiplexing conditions of 98 °C for 30 s and 32 cycles of 98 °C for 10 s, 54 °C for 30 s, 72 °C for 45 s, and then 72 °C for 10 min. Ultrapure water was used as a negative control to monitor the false-positive results of the PCR. The amplified product was further purified using AMPure XT beads (Beckman Coulter Genomics, Brea, CA, USA) to obtain the amplicon pools, which were then assessed using Agilent 2100 Bioanalyzer (Agilent, Santa Clara, CA, USA) and quantified using the Library Quantification Kit for Illumina (Kapa Biosciences, Wilmington, MA, USA). The sequencing of the libraries was performed on the Illumina NovaSeq PE250 platform (250 base pair × 250 base pair).

### 2.5. Bioinformatics Analysis

High-quality clean tags were obtained by screening the raw FLASH-merged paired-end reads using fqtrim (v0.94). Chimeric sequences were filtered using the Vsearch software (v2.3.4), and dereplication was performed using DADA2 to obtain feature sequences of the fecal samples. Then, each representative nucleotide sequence was aligned with BLAST and the SILVA ribosomal RNA database (http://www.arb-silva.de/, accessed on 12 March 2020) for taxonomic classification. Alpha (α) diversity and beta (β) diversity were analyzed using QIIME2 (v1.8.0) and the SILVA (release 132) classifier. The feature abundance was normalized based on the relative abundance of each specimen. To assess the significance of β-diversity, PCoA analysis with an ANOSIM test was conducted. The quantification of β-diversity involved the utilization of the weighted UniFrac method for distance calculation. BUGbase analysis was performed to predict bacterial phenotypes using the BugBase website (https://github.com/knights-lab/BugBase, accessed on 12 March 2020). The linear discriminant analysis (LDA) effect size (LEfSe) was assessed using the nsegata-lefse software (v094f447691f0) with LDA > 4.0. Metagenome functions were predicted using PICRUSt2 (Phylogenetic Investigation of Communities by Reconstruction of Unobserved States) with picrust2.2.0b. SparCC correlation analysis was performed using the FastSpar software (v0.0.10). The data were visualized using the R package (v3.4.4) with ggalluvial for the Sankey diagram and corrplot for the correlation heatmap.

### 2.6. Statistical Analysis

Graphpad (v8.0) was employed to generate statistical diagrams. The Wilcoxon rank sum test was applied to compare the distributions of continuous variables in two unpaired groups, while the Wilcoxon signed rank test was used for paired groups. The proportions of categorical variables were compared using Fisher’s exact test. *p* < 0.05 was considered statistically significant for the above tests. The relative abundances of the taxa of the gut microbiota were compared using adjusted multiple comparisons via the Benjamini–Hochberg (BH) correction (adjusted *p ≤* 0.15) [17]. 

## 3. Results

The demographic information of all participants is presented in the Supplementary Information—Materials and Methods (Table S1). A total of 75 collected fecal samples were amplified via 16S rRNA PCR, and they were sequenced and analyzed with the following purposes.

### 3.1. Similar Microbial Abundance within the LD and ESRD Fecal Specimens

**Figure 2:**
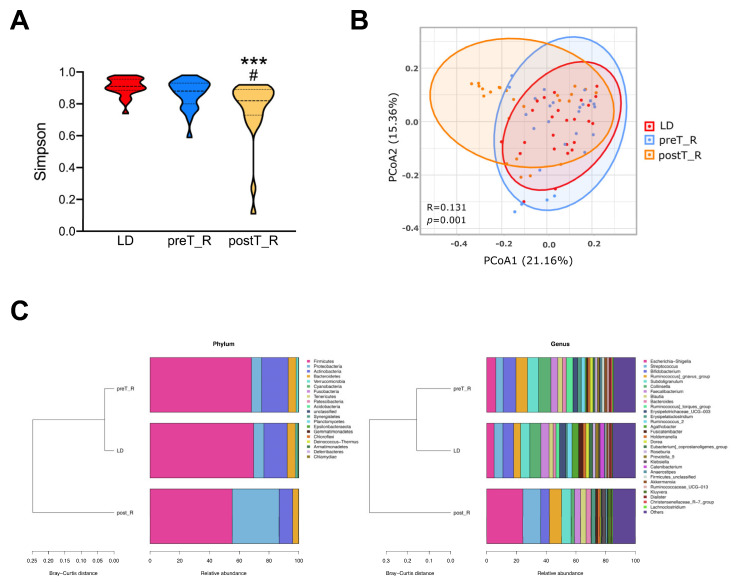
Diversity and composition of fecal microbiota. (**A**) Simpson α−diversity index in the LD, preT_R, and postT_R groups. No notable differences in fecal microbial diversity were observed between the healthy LDs and ESRD pre−transplant recipients. Instead, a significant decrease in microbial diversity was evident after kidney transplantation. *** *p* < 0.001 vs. LD group; ^#^ *p* < 0.05 vs. preT_R group. (**B**) The β−diversity according to the weighted principal coordinate analysis (PCoA) revealed significant intergroup differences among the three groups; (**C**) relative taxonomic abundance of fecal microorganisms at the phylum level (**left** panel) and genus level (**right** panel) among the three groups.

ESRD is commonly associated with gut dysbiosis due to metabolic disorder and immune dysregulation. Surprisingly, little difference in the intestinal microbial composition was found in *Cohort 1* between the LDs and paired preT_R ESRD participants. There was also no significant difference in the microbial richness, as measured according to the mean (±SD) Simpson diversity index (LD 0.91 ± 0.06, preT_R 0.86 ± 0.09, *p* = 0.08, Figure 2A), as well as other α-diversity indices (Figure S1). Additionally, no statistically significant difference in β-diversity was observed between these two groups via PCoA analysis (R = −0.014, *p* > 0.05, Figure 2B and Figure S2).

An increased relative abundance of *Actinobacteria* and a decreased relative abundance of *Firmicutes* at the phylum level, along with decreased relative abundances of *Agathobacter*, *Catenibacterium*, *Fusicatenibacter* and *Eubacterium* and increased relative abundances of *Ruminococcus]_gnavus_group*, *Ruminococcus]_torques_group*, *Erysipelatoclostridium*, *Subdoligranulum*, *Bifidobacterium,* and *Escherichia–Shigella* at the genus level, were observed in the preT_R patients compared to the healthy controls (relative abundance difference > 1%, Figure 2C, Table S2), although these differences were not statistically significant (BH-adjusted *p* > 0.15).

### 3.2. Changes in the Microbial Population Associated with Kidney Transplant

We next compared the alterations in the intestinal microbiota following KT in *Cohort 2*. The Simpson diversity index was significantly lower in the postT_R group than in the preT_R group (0.77 ± 0.19 vs. 0.86 ± 0.09, *p* = 0.01). Meanwhile, the structure of the microbial community after KT was significantly distinct from that before surgery (PCoA analysis: R = 0.170, *p* = 0.001, Figure 2A,B and Figures S1 and S2).

**Figure 3:**
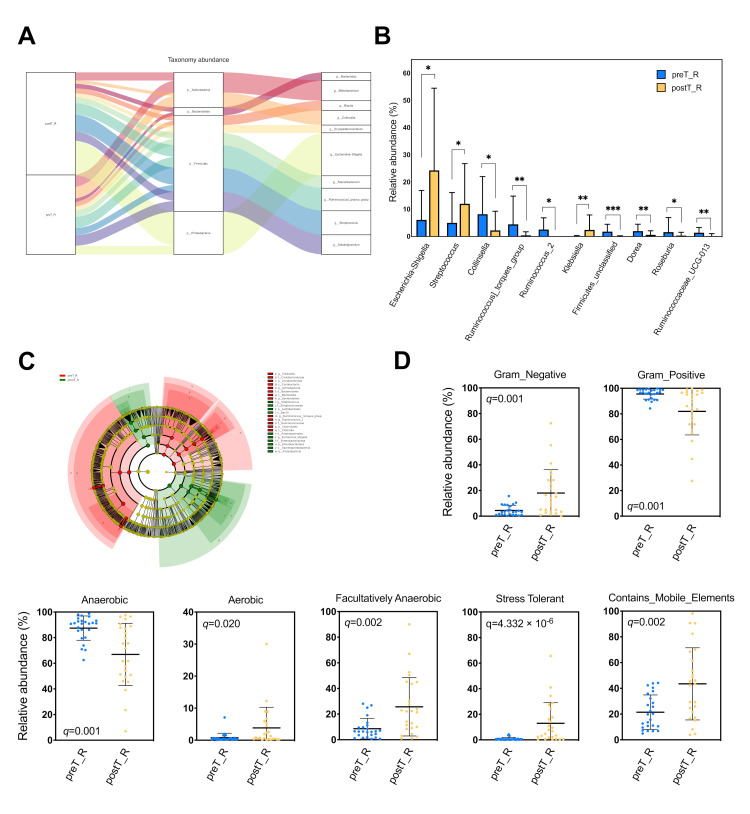
Gut dysbiosis in allograft recipients after kidney transplantation. (**A**) Sankey plot illustrating the correspondence of major gut microbiota between the preT_R and postT_R groups (left), annotated at the phylum level (medium) and genus level (right). The width of the flow represents the relative proportions. (**B**) Column plot displaying the significantly different prominent taxa at the genus level between the preT_R and postT_R groups, with a Benjamini–Hochberg (BH)-adjusted *p* of <0.15 and an absolute abundance difference of >1.0. Data are presented as mean ± SD. * BH-adjusted *p* < 0.15, ** BH-adjusted *p* < 0.05, *** BH-adjusted *p* < 0.01. (**C**) Cladogram demonstrating crucial microbiota taxa with a phylogenetic relationship using an LDA effect size (LEfSe) analysis. Each circle represents a taxonomic classification from the phylum to the species level from the inside to the outside. Yellow nodes represent bacterial taxa with no significant differences in relative abundance between the two groups, while red nodes and green nodes indicate greater enrichment in the preT_R and postT_R groups, respectively, on the basis of LDA > 4. *p*, phylum; *c*, class; *o*, order; *f*, family; *g*, genus. (**D**) Relative abundance of Gram-negative, Gram-positive, anaerobic, aerobic, facultatively anaerobic, and stress-tolerant bacteria, as well as those containing mobile elements, according to the BUGbase analysis. Data are presented as mean ± SD. The *q*-value denotes the BH-adjusted *p*-value.

The fecal microbiota of the recipients were dominated by two separate bacterial phyla: *Firmicutes* (68.2%) and *Actinobacteria* (17.8%) in preT_R versus *Firmicutes* (55.2%) and *Proteobacteria* (31.6%) in postT_R. The increase in *Proteobacteria* was largely due to an enrichment of *Escherichia–Shigella*, which belongs to the *Enterobacteriales* order, while the decrease in *Actinobacteria* corresponded mainly with a reduction in *Collinsella* from the *Coriobacteriales* order (Figure 2C and Figure 3A).

At the genus level, a total of 69 bacterial genera were significantly different in terms of relative abundance between the paired pre-transplant and post-transplant fecal specimens (BH-adjusted *p* ≤ 0.15). Notably, there was nearly a four-fold increase in the relative abundance of *Escherichia–Shigella* (6.10% to 24.29%, BH-adjusted *p* = 0.04) and a 2.5-fold increase in *Streptococcus* (5.02% to 12.02%, BH-adjusted *p* = 0.10); meanwhile, there was a 3.5-fold reduction in the relative abundance of *Collinsella* (8.20% to 2.26%, BH-adjusted *p* = 0.07) in the fecal samples from recipients after transplantation compared to their pre-transplant specimens. Furthermore, the relative abundance of *Klebsiella* (0.09% to 2.44%, BH-adjusted *p* = 0.05) increased from a very low level, whereas that of *Ruminococcus_2* (2.57% to 0.01%, BH-adjusted *p* = 0.04) and *Ruminococcus_torques_group* (4.46% to 0.44%, BH-adjusted *p* = 0.07) descended to a low level after KT (Figure 3B). The detailed data of the prominent taxa in these two groups—with an absolute abundance difference larger than 1.0—are shown in Table 1.

**Figure 4:**
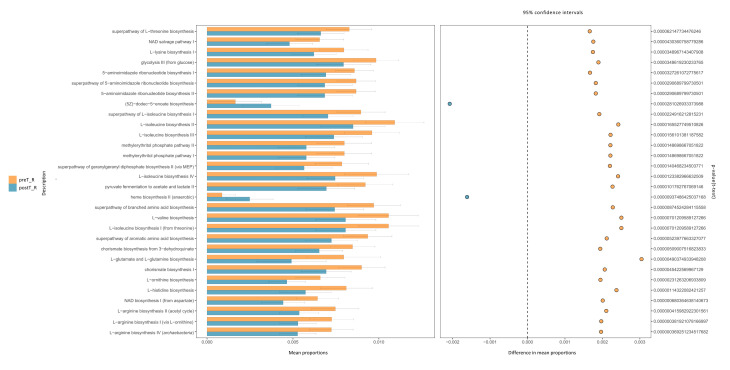
Metabolic pathways distinguishing the fecal specimens after kidney transplantation. PICRUSt2 analysis revealing the top 30 significantly different KEGG functional pathways between the preT_R and postT_R groups (with the minimum BH-adjusted *p* values). The relative mean abundances of these functional pathways, with the preT_R group depicted in orange and the postT_R group in blue (**left** panel). The mean differences in KEGG pathways, with orange points representing a significantly higher abundance in the preT_R group and blue points representing a significantly higher abundance in the postT_R group (**right** panel).

A supervised phylogenetic distribution analysis was conducted via LEfSe (Figure 3C). The BUGbase analysis indicated that the relative abundance of Gram-negative bacteria, aerobic bacteria, facultatively anaerobic bacteria, stress-tolerant bacteria, and those containing mobile elements significantly increased, while the relative abundance of Gram-positive bacteria and anaerobic bacteria significantly decreased (Figure 3D). We utilized PICRUSt2 to predict the metagenome functions in the pre-transplant and post-transplant gut specimens, and the top 30 significantly different KEGG pathways with minimal *p*-values were identified (Figure 4).

### 3.3. Bacterial Taxa Associated with Early Rejection Episodes

We further compared the differences in the gut microbiota of the post-transplant rectal samples from the postT_R_NB group (*n* = 23) with those from the postT_R_BL group (*n* = 2) in this Sub-cohort study. A similar microbial richness was identified between these two groups (Simpson diversity index: postT_R_NB 0.76 ± 0.20, postT_R_BL 0.87 ± 0.07, *p* = 0.36, Figure S3).

**Figure 5:**
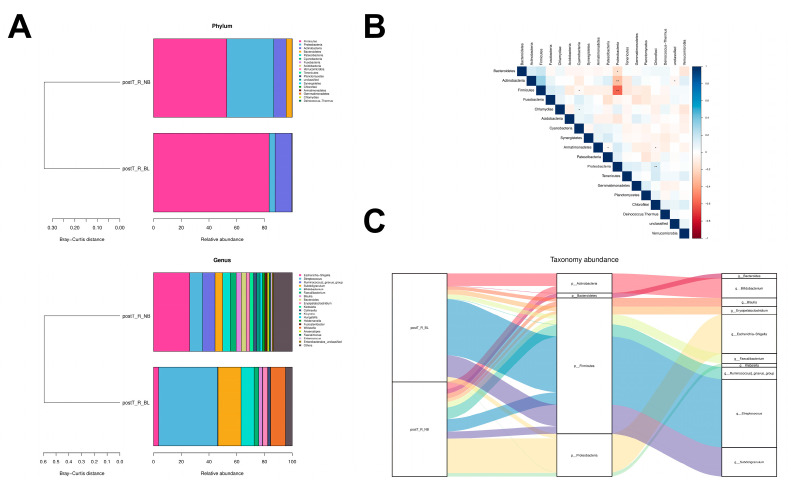
Gut bacterial alterations in patients with biopsy−proven borderline episodes. (**A**) Relative taxonomic abundance of fecal microorganisms at the phylum level (upper panel) and genus level (lower panel) between the postT_R specimens (postT_R_BL) and the non−BL postT_R specimens (postT_R_NB) with biopsy−proven borderline change (BL). (**B**) SparCC correlation heatmap matrix with the significance level expressed by asterisks (* *p* < 0.05, ** *p* < 0.01). The intensity of the color represents the correlation coefficient. (**C**) Sankey plot displaying the correspondence of major gut microbiota between the postT_R_NB and postT_R_BL groups (left), annotated at the phylum level (medium) and genus level (right). Diverse colors represent distinct corresponding genera, while the width of the flow represents the relative proportions of the identified microbiota.

In the biopsy-proven BL patients, a striking expansion of *Firmicutes* (52.78% to 83.34%) and an evident decrease in *Proteobacteria* (33.92% to 4.31%) at the phylum level were observed compared to the non-BL patients, along with an apparent negative correlation (ρ = −0.59, *p* = 0.009, Figure 5A,B). The Sankey plots showed that the elevated relative abundance of *Firmicutes* was mostly contributed by the increases in *Streptococcus* (9.37% to 42.55%, BH-adjusted *p* = 0.76) and *Subdoligranulum* (5.54% to 16.58%, BH-adjusted *p* = 0.82), while the decreasing relative abundance of *Proteobacteria* was mainly due to the reductions in *Escherichia–Shigella* (26.09% to 3.68%, BH-adjusted *p* = 0.82) and *Klebsiella* (2.69% to 0.16%, BH-adjusted *p* = 0.82) at the genus level, despite the statistical insignificance (Figure 5C, Table S3). Only six bacterial genera reached a significant difference in relative abundance between the non-BL and BL fecal specimens (BH-adjusted *p* ≤ 0.15), including the coincident slight increases in *Curtobacterium* (0.000% to 0.037%, BH-adjusted *p* = 0.04), *Flexilinea* (0.000% to 0.004%, BH-adjusted *p* = 0.04), *Enorma* (0.000% to 0.003%, BH-adjusted *p* = 0.04), *Coprococcus_2* (0.000% to 0.002%, BH-adjusted *p* = 0.04), *Muribaculum* (0.000% to 0.002%, BH-adjusted *p* = 0.04), and *Planctopirus* (0.000% to 0.002%, BH-adjusted *p* = 0.04).

## 4. Discussion

The comprehensive characterization of microbial population shifts during chronic kidney disease and KT holds the potential to enhance our understanding of disease-related intestinal dysbacteria and facilitate the identification of predictive microbial taxa associated with clinical etiology. Our findings revealed a comparable construction of the gut microbiota between ESRD patients and their healthy relatives, while KT exerted a substantial impact on the composition of the gut microbiota. 

In *Cohort 1*, we conducted a comparison of the gut microbial communities between the ESRD individuals, who were primed for KT surgery, and their healthy relatives as living donors. Multiple synergistic factors participate in the development and remolding of mammalian intestinal microbiomes. Pathological accumulation of uremic toxins in the gastrointestinal tract is presumed to exert an influence on the gut microorganism composition in ESRD patients, leading to a contraction of short-chain fatty acid (SCFA)-producing bacteria and an expansion of indole-forming and p-cresol-forming bacteria [20]. Vaziri et al. identified 190 bacterial taxa (e.g., *Brachybacterium*, *Catenibacterium*, *Enterobacteriacea*, etc. at the family level) with a markedly elevated abundance (BH-adjusted *p* < 0.02) in 24 ESRD patients compared to 12 healthy individuals [21]. Feiqian et al. reported an overgrowth of *Klebsiella*, *Proteus*, *Escherichia*, *Enterobacter*, and *Pseudomonas* at the genus level in non-dialyzed ESRD patients (*n* = 30) when contrasted with healthy controls (*n* = 10), with unadjusted *p* < 0.05 being considered statistically significant [22]. However, it is important to acknowledge that the enrollment of randomly selected healthy individuals as controls in the aforementioned studies may have introduced inter-individual variations beyond the impact of gut toxin accumulation. Recent research has underscored the influence of genetics [23,24] and diet [20] on shaping the structure of the mammalian intestinal microbiome. For example, host SNPs in mucin-encoding genes are correlated with the relative abundance of mucin-degrading gut bacteria, which are involved in the host’s health and nutritional acquisition [25]; in addition, enterotypes from a long-term diet of animal fat and protein are distinguished primarily by *Bacteroides*, while carbohydrates are distinguished by *Prevotella* [26]. Therefore, we made a preliminary attempt to homogenize the diet composition and genetic background by enrolling living donors from the same family as the ESRD patients, assuming that these participants would likely share similar food preferences and genotypes. Among the living kidney donors in our study, 22 (88%) were from parents and given to children, 1 (4%) was from a biological sister, and 2 (8%) were from married couples. Our study found no significant differences in the relative richness of enterotypes between ESRD and healthy groups, which was consistent with the report by Vaziri et al. [21]. Additionally, no substantial differences in the relative abundance of gut microbial populations were observed in our study, contrary to the results of the aforementioned studies. This discrepancy could potentially be attributed to the relatively minor differences in the host genetic and/or dietary factors within our study cohort. 

The application of perioperative antibiotics and high-dose immunosuppression in KT has been recognized as a contributing factor to gut bacterial dysbiosis. However, it is worth noting that certain studies have encountered challenges with sample collection. For instance, the investigation by Fricke et al. experienced issues with ineffective sampling, resulting in a loss of more than 50% of the participants within one month after KT [10]. To address this, we longitudinally compared the alterations in gut microbial communities following KT in *Cohort 2* from immediately before the surgery to approximately 2 months post-transplantation, without any patients being lost to follow-up. Consistently with previous observations [7,10,12], a decreased microbial richness measured with the α-diversity index was observed after KT, which was probably due to the administration of preventive antimicrobial medications, such as cefuroxime sodium or levofloxacin, during the perioperative period. However, Lee et al. did not observe a significant reduction in microbial diversity after KT, which may be attributed to the smaller sample size (*n* = 5) and shorter follow-up time (2 weeks) [11]. Despite this, the reduced diversity of the microbiota after KT resulted in the dominance of two bacterial taxa at the genus level, namely, *Escherichia–Shigella* and *Streptococcus*, which constituted nearly 24% and 12% of the entire gut flora, respectively. Even 1.5 years after KT, the relative abundance of *Streptococcus thermophilus* and *Escherichia coli* at the species level remained significantly higher compared to that found in healthy controls [7]. As is well known, *Escherichia–Shigella* from the *Proteobacteria* phylum is a common pathogen that causes urinary tract infections. Taur et al. reported a five-fold-increased risk of bacteremia when the relative abundance of fecal *Proteobacteria* exceeded 30% [27]. Lee et al. found a higher relative abundance of fecal *Escherichia* in recipients with post-transplant diarrhea than in the no-diarrhea group [17]. During our study period, opportunistic infections were not observed. Therefore, we did not investigate the relationships between specific fecal taxa and infectious complications. Further studies are needed to determine the exact role and mechanisms in this context.

BUGbase analysis was then performed to characterize the specific features of gut microbial taxa with significantly different relative abundances. The results revealed a consistent upregulation in the relative abundance of Gram-negative *Escherichia–Shigella*, Gram-positive *Streptococcus*, and facultatively anaerobic and stress-tolerant bacteria in allograft recipients following KT. Conversely, a consistent downregulation was observed in the relative abundance of *Collinsella*, along with Gram-positive and anaerobic bacteria. To gain insight into the microbial functional potential [28], we applied PICRUSt2 analysis to infer the presence of specific gene families and metabolic pathways. This analysis revealed the significant upregulation of (5Z)-dodec-5-enoate biosynthesis and the heme biosynthesis II (anaerobic) pathway in the intestinal microorganism community after KT. *Escherichia–Shigella* is well known for its diverse metabolic capabilities, including heme acquisition from the host [29]; however, the specific mechanisms involved in the anaerobic heme biosynthesis pathway in *Escherichia–Shigella* or other gut bacteria remain to be fully elucidated. 

Studies exploring the intricate interplay between gut microorganisms and renal graft rejection episodes are limited, and findings may vary across studies. Fricke et al. conducted a study comparing the gut microbial compositions of four patients experiencing later rejection events with 14 stable recipients using their pre-transplant rectal samples. They revealed significant decreases in *Anaerotruncus*, *Coprobacillus*, *Coprococcus,* and an unknown member of the *Peptostreptococcaceae* genus (all from the Firmicutes phylum, *p* < 0.005) [10]. However, it is crucial to note that the abundance of gut microbiota can undergo substantial variations after KT, as observed in previous research and our *Cohort 2* findings. Therefore, caution should be exercised when interpreting findings based on pre-transplant specimens. Lee et al. investigated the post-transplant fecal samples of three recipients experiencing acute rejection and 23 recipients without rejection, revealing a significantly lower relative abundance of the *Bacteroidetes* phylum and *Clostridiales* order, as well as a higher relative abundance of *Lactobacillales* order (*p* < 0.05) [11]. However, potential confounding factors arose due to the administration of multiple antibiotics during their sample collection. Borderline change (BL) for acute TCMR is an episode of allograft early rejection. In our preliminary study, we enrolled a Sub-cohort to compare the post-transplant fecal specimens of two recipients who were subsequently proven to have BL within 6 months after KT via biopsy with the remaining non-BL samples. Our findings revealed a striking increase in the prevalence of the *Streptococcus* and *Subdoligranulum* genera within the *Firmicutes* phylum, as well as a decrease in *Escherichia–Shigella* and *Klebsiella* within the *Proteobacteria* phylum; however, the difference did not reach statistical significance, which was possibly due to the small sample size of BL recipients and the stringent statistical standard, as a BH-adjusted *p*-value threshold of less than 0.05 was utilized, while several previous studies have used a non-adjusted *p*-value as the statistical parameter [11,30]. Thus, only six bacterial genera exhibited statistically significant differences in relative abundance, and the observed variations were relatively limited in our study. However, in recipients with ABMR, a much greater number of significantly different gut genera, including *Curtobacterium*, were observed compared to those with stable post-transplant renal functions. Furthermore, the correlated alterations in fecal microbial metabolites were found to have the potential to serve as diagnostic biomarkers [30]. Given the distinct mechanisms of ABMR and TCMR, there is a need for integrative metagenomic and metabolomic analyses that can reveal differences between these rejection types. 

Our study contributed to the growing body of knowledge regarding the complex relationship among the gut microbiota, ESRD, and transplantation. However, there were certain limitations to this study. Firstly, the sample size was relatively small, which constrained our ability to delve deeper into the potential mechanisms underlying the similarity in the composition of the gut microbiome between ESRD patients and their healthy relatives, particularly in relation to dietary habits and genetic backgrounds. Secondly, we did not investigate the complex interplay among gut microorganisms, microbial metabolites, and the host’s metabolic processes, which represents an essential aspect of this field. Consequently, further research with a larger sample size and a more rigorous study design is imperative to comprehensively unravel the intricate mechanisms that connect the gut microbiome, metabolomics, immune responses, and renal graft outcomes.

## 5. Conclusions

In summary, our study found no significant difference in the composition of the gut microbiome between East Asian ESRD patients and their healthy relatives. However, following KT, intestinal dysbacteria developed, which was characterized by decreased microbial diversity and increased relative abundances of *Escherichia–Shigella* and *Streptococcus*. Moreover, our preliminary findings in the Sub-cohort indicated potential shifts in microbial composition during early rejection episodes. To gain a comprehensive understanding of the intricate relationship among the gut microbiome, immune responses, and renal graft outcomes, further research with larger sample sizes and rigorous study designs is necessary.

## Figures and Tables

**Table 1:** Relative abundance of the prominent gut microbiota in the preT_R and postT_R groups.

	Mean Relative Abundance (%)			BH-Adjusted *p*-Value
	preT_R	postT_R	Difference	
Phylum level				
*p__Proteobacteria*	6.83	31.54	24.71	0.01
*p__Actinobacteria*	17.89	9.18	8.71	0.05
*p__Bacteroidetes*	5.26	3.92	1.34	0.05
Genus level				
*g__Escherichia-Shigella*	6.10	24.29	18.19	0.06
*g__Streptococcus*	5.02	12.02	7.01	0.07
*g__Collinsella*	8.20	2.26	5.94	0.08
*g__Ruminococcus_torques_group*	4.46	0.44	4.01	0.02
*g__Ruminococcus_2*	2.57	0.01	2.56	0.12
*g__Klebsiella*	0.09	2.44	2.35	0.03
*g__Firmicutes_unclassified*	1.81	0.09	1.72	0.00
*g__Dorea*	1.97	0.63	1.34	0.05
*g__Roseburia*	1.63	0.36	1.26	0.14
*g__Ruminococcaceae_UCG-013*	1.40	0.20	1.20	0.04

## Data Availability

Data are partially available in the Supplementary Files.

## Supplementary Materials

The following supporting information can be downloaded at: https://www.mdpi.com/article/10.3390/microorganisms11112747/s1. Figure S1. Violin graph of α-diversity within three groups; Figure S2. β-diversity of the gut microbial community via PCoA analysis; Figure S3. Diversity of the gut microbial community in graft recipients with or without borderline change (BL); Table S1. Demographic and clinicopathological information; Table S2. Relative abundance of the gut microbial community in the LD and preT_R groups; Table S3. Relative abundance of the gut microbial community in the postT_R_NB and postT_R_BL groups.
