# Supplementary material for: Unraveling Intestinal Microbial Shifts in ESRD and Kidney Transplantation: Implications for Disease-Related Dysbiosis
Source: Microorganisms. 2023 Nov 10;11(11):2747. doi: 10.3390/microorganisms11112747 (PMC10673061; doi:10.3390/microorganisms11112747)
Supplement: Supplementary file 1 [file microorganisms-11-02747-s001.zip › microorganisms-2601524-supplementary.pdf]

Supplemental materials

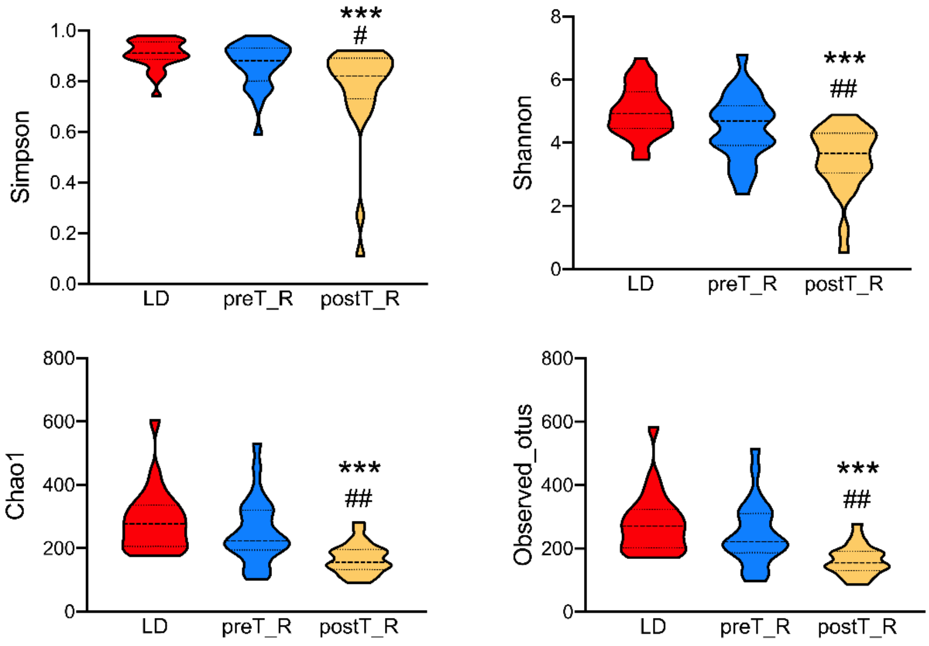

Figure S1. Violin graph of α-diversity within three groups

The α-diversity indices calculated for the Observed_otus, Chao1, Shannon and Simpson indices among three groups. The comparison revealed no significant difference between the LD group and preT_R group ($p > 0.05$, Kruskal-Wallis test), while each index was significantly down-regulated in the postT_R group compared to both the LD group and preT_R group. $^{***}p < 0.001$ vs LD group； $^{\#\#}p < 0.01$ vs preT_R group， $^{\#}p < 0.05$ vs preT_R group.

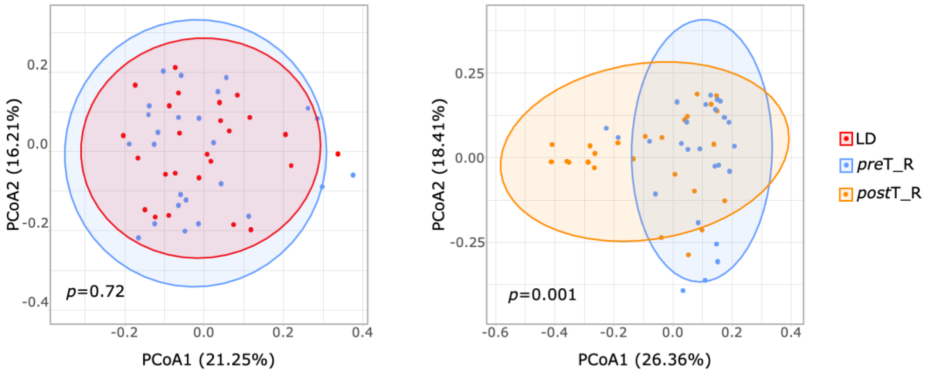

Figure S2. β-diversity of gut microbiota community via PCoA analysis

Weighed PCoA revealed similar community structures between the healthy living donors (LD) and the pre-transplant ESRD recipients (preT_R) groups, where the first two principal components represented 21.25% and 16.21% of the total variations (left panel). However, a distinct microbial structure was observed in the postT_R group compared to the preT_R gourp, with the first two principal components accounting for 26.36% and 18.41% (right panel). Symmetrical distribution of fecal microorganisms was identified between the LD and preT_R groups (p = 0.72, ANOSIM test with weighted Spearman rank analysis), while a markedly different distribution was confirmed between preT_R and postT_R (p = 0.001, ANOSIM test with weighted Spearman rank analysis).

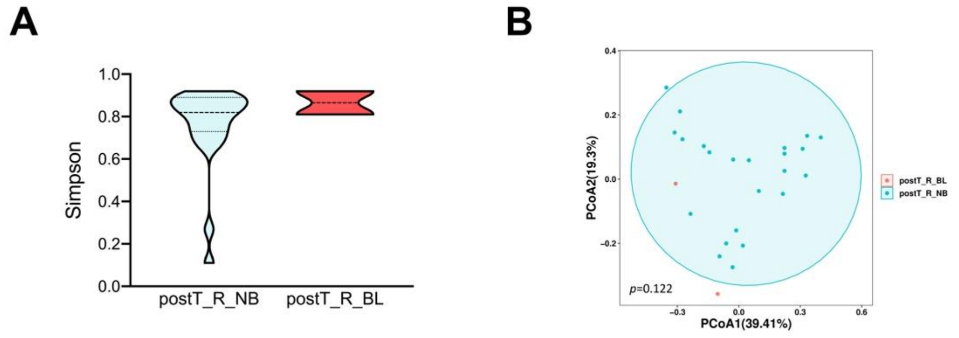

Figure S3. Diversity of gut microbiota community in graft recipients with or without borderline change (BL)

No significant difference in α-diversity were identified, as measured by the Simpson index between the postT_R_NB group (n = 23, 0.76 ± 0.20) and the postT_R_BL group (n = 2, 0.87 ± 0.07) (p = 0.357, **A**). Similarly, β-diversity assessed by PCoA analysis also showed no significant distinction between the two groups (p = 0.122, **B**).

Table S1. Demographic and clinicopathological information

	Living Donors (n = 25)	ESRD Recipients (n = 25)
Age	54.2±4.9	32.4±8.6
Female, N (%)	21 (96%)	7 (28%)
Serum creatitine (μmol/L)	59.1±10.3	930.0±259.6
BUN(mmol/L)	5.3±1.2	19.4±6.7
Uric acid (μmol/L)	251.8±61.8	390.2±118.9
History of hypertension, N (%)	6 (24%)	25 (100%)
History of diabetes mellitus, N (%)	0	0
BMI (kg/m^2)	23.0±2.6	21.1±4.0
BMI≥28kg/m^2, N (%)	1	2
BMI<18.5kg/m^2, N (%)	0	5
CKD duration (month）		24 [10,60]
RRT duration (month）		2.6 [1.8,4.0]
RRT methods		
Hemodialysis (%)		17 (68%)
Peritoneal Dialysis (%)		4 (16%)
none (%)		4 (16%)
Biological relationship		
Parents to children, N (%)	22 (88%)	
Married couples, N (%)	2 (8%)	
Biological sisters, N (%)	1 (4%)	

Data are shown in mean±SD, except the RRT and CKD duration are shown in median [lower quartile, upper quartile] .CKD, chronic kidney disease; ESRD, end-stage renal disease; BUN, blood urea nitrogen; BMI, body mass index; RRT, renal replacement therapy.

Table S2. Relative abundance of gut microbiota community in the LD and preT_R group

	Mean relative abundance (%)			BH adjusted p value
	LD	preT_R	Difference	
**Phylum level**				
Actinobacteria	15.69	17.89	2.20	0.91
Firmicutes	69.69	68.19	1.49	0.95
**Genus level**				
g__Agathobacter	4.48	0.92	3.56	0.50
g__Ruminococcus]_gnavus_group	4.63	7.80	3.17	0.53
g__Catenibacterium	2.91	0.31	2.60	0.53
g__Ruminococcus]_torques_group	2.42	4.46	2.04	0.99
g__Fusicatenibacter	2.93	0.94	1.99	0.50
g__Erysipelatoclostridium	1.08	2.48	1.40	0.53
g__Subdoligranulum	6.17	7.48	1.31	0.53
g__Bifidobacterium	7.27	8.50	1.24	0.60
g__Escherichia-Shigella	4.96	6.10	1.15	0.57
g__Eubacterium]_coprostanoligenes_group	2.42	1.36	1.07	0.53

Table S3. Relative abundance of gut microbiota community in the postT_R_NB and postT_R_BL group

	Mean relative abundance (%)			BH adjusted *p* value
	postT_R_NB	postT_R_BL	Difference	
**Phylum level**				
p__Firmicutes	52.78	83.34	30.56	0.77
p__Proteobacteria	33.92	4.31	29.62	0.77
p__Bacteroidetes	4.23	0.31	3.92	0.89
p__Actinobacteria	8.92	11.98	3.06	0.81
**Genus level**				
g__Streptococcus	9.37	42.55	33.18	0.76
g__Escherichia-Shigella	26.09	3.68	22.40	0.82
g__Subdoligranulum	5.54	16.58	11.04	0.82
g__Weissella	0.37	10.66	10.29	0.62
g__Ruminococcus]_gnavus_group	8.89	0.29	8.60	0.82
g__Bifidobacterium	5.49	9.41	3.92	0.82
g__Bacteroides	3.54	0.22	3.31	0.82
g__Klebsiella	2.69	0.16	2.53	0.82
g__Kluyvera	2.40	0.01	2.40	0.82
g__Hungatella	1.72	0.00	1.72	0.82
g__Holdemanella	1.67	0.00	1.67	0.82
g__Fusicatenibacter	1.46	0.13	1.33	0.82
g__Anaerostipes	1.27	0.00	1.27	0.82
g__Faecalimonas	1.12	0.00	1.12	0.82
g__Enterococcus	1.07	0.01	1.07	0.82
